# Gangrene of the Buccal Mucous Membrane Caused by Cold

**Published:** 1858-07

**Authors:** 


*Gangrene of the Buccal Mucous Membrane caused by cold.*—The following case is reported by Dr. Chaloin:

X——, aged fifty-nine years, having gone out to cut wood, on the 27th of January, 1855, was obliged to break a path through the snow. Heated by this labor, he sought to quench his thirst by occasionally allowing morsels of ice to melt in his mouth. The next day he had swelling of the mucous membrane, of which the portion covering the interior of the lower jaw was completely detached, and came up to the level of the teeth. It was of a deep red color and a fungous appearance. The expression was one of deep anxiety; the extremities were cold; there was delirium during sleep, and the pulse beat a hundred a minute. Scarifications in the mucous membrane gave issue to a black blood of an offensive odor. The part was cauterized with nitrate of silver, twenty leeches applied to the lower jaw, chlorinated gargles, with decoctions of bark, were used, and dry frictions applied to the upper extremities. Sinapisms were also employed. It was impossible to get the smallest drop of liquid inside the mouth. Five hours afterward the fever ceased, a strong gangrenous odor was perceived, and the patient soon died.—*L'Art Dentaire.*

